# Adult Children’s Education Attainment and Parents’ Subjective Well-Being in China

**DOI:** 10.1093/geroni/igaa057.1104

**Published:** 2020-12-16

**Authors:** Dan Chen, Yuying Tong

**Affiliations:** The Chinese University of Hong Kong, Hong Kong, China

## Abstract

Parent-child tie is important for parental later life due to insufficient old-age support in developing contexts. Parents often anticipate they would share the returns of children’s education for their early period investment. Previous studies show that adult children’s education is positively associated with parents’ survival and physical health in both low- and middle-income countries. What’s less discussed in literatures is the effect of adult children’s education on parental subjective wellbeing. Drawing the China Health and Retirement Longitudinal Study (CHARLS), this study intends to explore the effect of adult children’s education attainment on parents’ life satisfaction. This study uses information from baseline wave in 2011 and latest wave in 2015 of CHARLS. The analytic sample restricts to adult children aged between 25 and 49 with the highest education among all children of a parent who are aged between 50 and 84. To handle the reversed causality, this study adopts lagged effect model and controls for baseline subjective wellbeing. Instrumental variables (IV) are also used to handle the endogeneity issue existing between children’s education and parental wellbeing to conclude a causal effect. The preliminary results without IV reveal that association between children’s schooling years and parents’ life satisfaction is non-linear. However, results with IV show that adult children’s schooling years are negative associated with parents’ life satisfaction. This study firstly draws attention on negative sides of children’s education attainment on parental subjective wellbeing which implies more studies to unfold the mechanisms underlying the association.

